# Supplementary material for: Acute EPA-induced learning and memory impairment in mice is prevented by DHA
Source: Nat Commun. 2020 Oct 29;11:5465. doi: 10.1038/s41467-020-19255-1 (PMC7596714; doi:10.1038/s41467-020-19255-1)
Supplement: Supplementary file 1 — Supplementary Information [file 41467_2020_19255_MOESM1_ESM.pdf]

Supplementary Information

Acute EPA-induced learning and memory impairment in mice is prevented by DHA

Ji-Hong Liu, Qian Wang, Qiang-Long You, Ze-Lin Li, Neng-Yuan Hu, Yan Wang, Zeng-Lin Jin, Shu-Ji Li, Xiao-Wen Li, Jian-Ming Yang, Xin-Hong Zhu, Yi-Fan Dai, Jiang-Ping Xu, Xiao-Chun Bai &Tian-Ming Gao*

Supplementary Figure 1

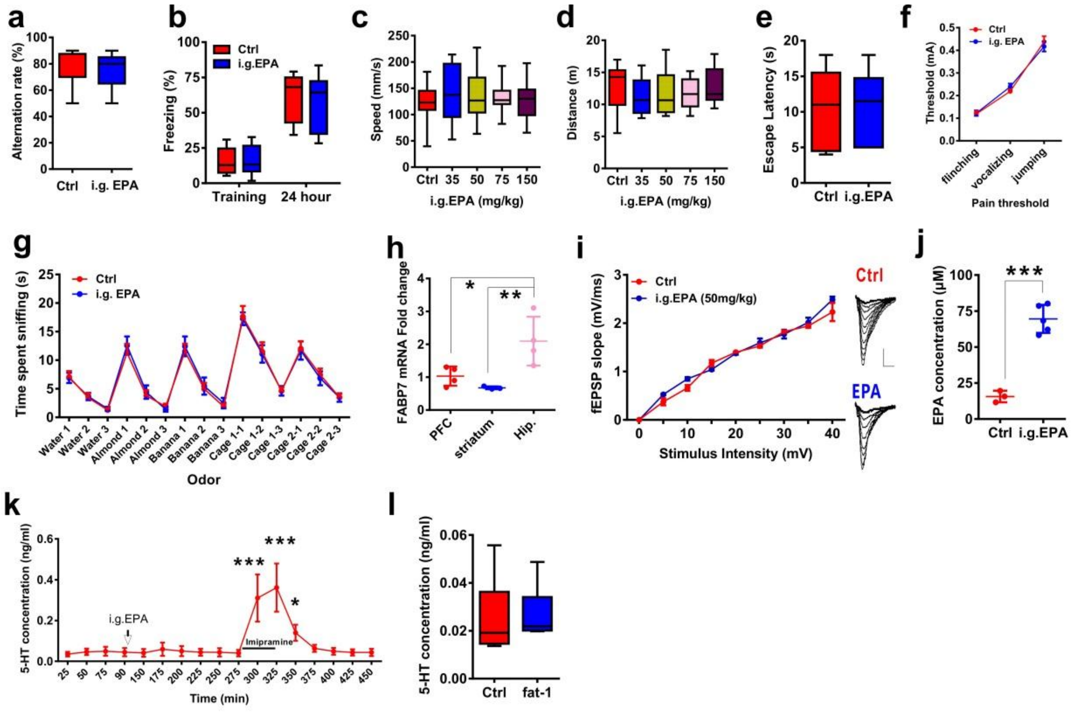

Supplementary Figure 1

Effects of i.g. administration of EPA on behaviours, synaptic transmission and hippocampal concentration of EPA and 5-HT.

(**a**) Alternation rate in the T-maze test (50 mg/kg, n=8,9 mice; two-tailed Student’s t-test, P=0.915).

(**b**) Freezing time in the cued fear conditioning test (50 mg/kg, n=8,9 mice/group; two-tailed Student’s t-test, P=0.541).

(**c**) Swimming speed in the MWM test (n=10,10,10,11,11 mice; one-way ANOVA, $F_{(4, 180)}$=10.219, P=0.927).

(**d**) Locomotor activity in the open field test (n=12,11,10,11,11 mice; one-way ANOVA, $F_{(4, 50)}$=15.516, P=0.724).

(**e-g**) No effect of i.g. administration of EPA (50 mg/kg) on sensory responses (n=8,9 mice; **e:** escape latency in the cued MWM test, two-tailed Student’s t-test, P=0.641; **f:** pain threshold in the fear conditioning test, repeated measures two-way ANOVA, $F_{(1, 51)}$=13.902, P=0.986; **g:** olfaction in the olfactory habituation/dishabituation test, repeated

measures two-way ANOVA, $F_{(1, 255)}$=17.129, P=0.873).

(**h**) FABP7 mRNA level in three brain regions (n=4 mice; one-way ANOVA, $F_{(2, 9)}$=37.284, P=0.026). PFC, prefrontal cortex; Hip., hippocampus.

(**i**) I-O curves after i.g. EPA (n=7 slices/group; repeated measures two-way ANOVA, $F_{(1, 108)}$=15.769, P=0.907).

(**j**) I.g. administration of EPA (50 mg/kg) increased the local EPA concentration in the hippocampus (n=5 mice/group; two-tailed Student's t-test, $P=0.56\times10^{-4}$).

(**k, l)** Microdialysis experiments: effect of i.g. EPA (**k**) and transgenic expression of fat-1 (**l**) on the extracellular 5-HT concentration in the hippocampus (**k**: n=6 mice; repeated measures one-way ANOVA, $F_{(23, 115)}$=58.668, $P=0.86\times10^{-3}$; **l**: n=6 mice/group; two-tailed Student's t-test, P=0.848).

Data show mean ± s.e.m. Scale bars: 0.5 mV, 5 ms. *P<0.05, **P< 0.01, ***P< 0.001.

Supplementary Figure 2

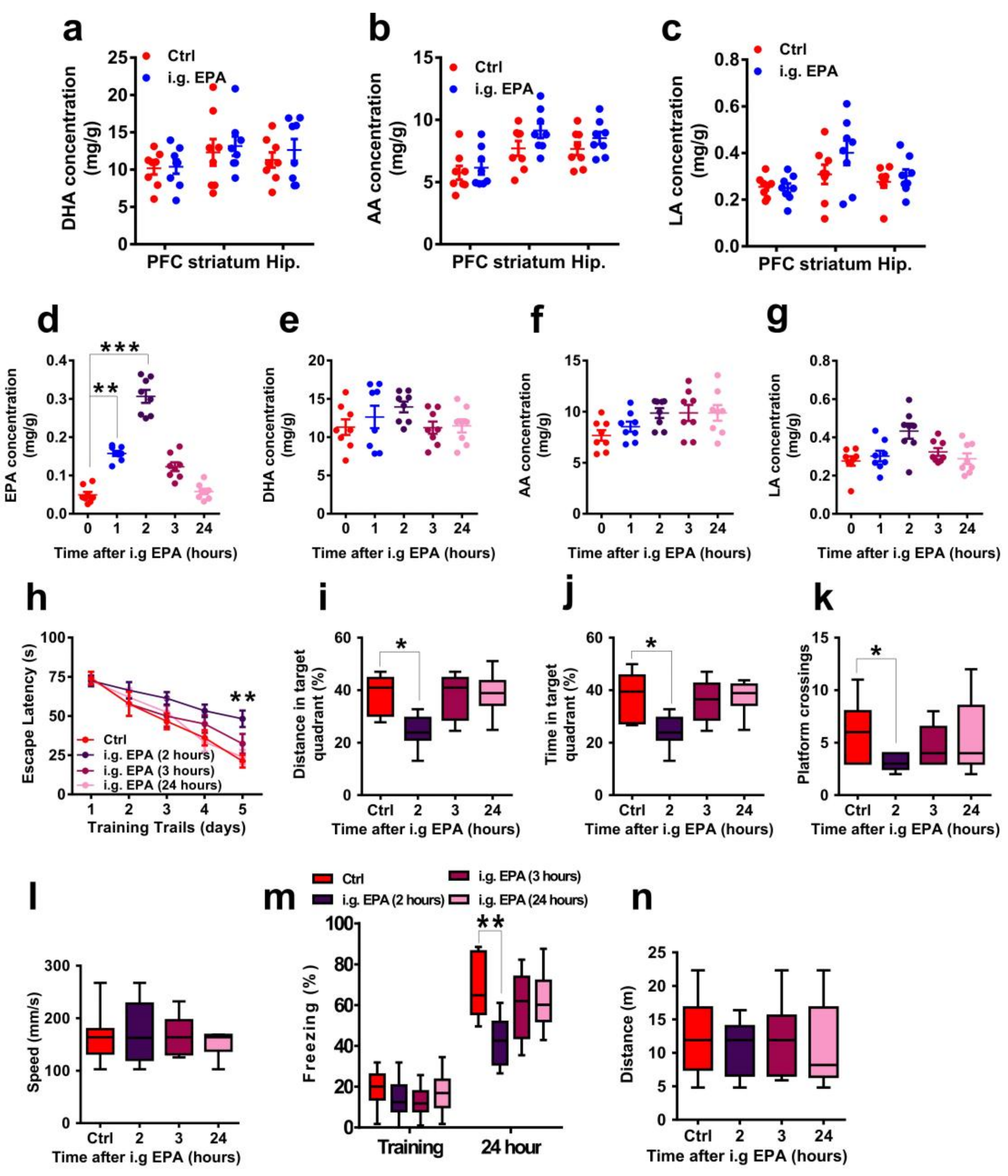

Supplementary Figure 2

Fatty acid levels in the brain and behaviors after i.g. administration of EPA.

(**a-c**) I.g. administration of EPA (50 mg/kg) did not affect DHA, AA, and LA levels in the brain (n =8 mice/group).

(**d-g**) The increase in the EPA level was time dependent in the hippocampus (n =8 mice/group; one-way ANOVA) (**d**, $F_{(4, 28)}$=40.924, P=0.007) but there was no change in DHA (**e**, $F_{(4, 28)}$=12.014, P=0.757), AA (**f**, $F_{(4, 28)}$=20.453, P=0.303),or LA (**g**, $F_{(4, 28)}$=17.268,

P=0.17) after i.g. EPA. AA, arachidonic acid; LA, linoleic acid.

(**h-n**) The impaired role of EPA on learning and memory was time dependent in MWM (n=9 mice/group; **h**: repeated measures two-way ANOVA, $F_{(3, 135)}$=42.468, P=0.013; * indicates differences between EPA [2 hours] and Ctrl [saline-treated]. **i-l**: one-way ANOVA; **i**: $F_{(3, 32)}$=38.643, P=0.022; **j**: $F_{(3, 32)}$=37.965, P=0.025; **k**: $F_{(3, 32)}$=39.361, P=0.021; **l**: $F_{(3, 32)}$=21.454, P=0.893) and contextual fear conditioning test (**m**: n=9 mice/group; one-way ANOVA; $F_{(3, 32)}$=38.616, P=0.005) without affecting the locomotor activity in the open field test (**n**: n=9 mice/group; one-way ANOVA; $F_{(3, 32)}$=25.479, P=0.732).

Data show mean ± s.e.m. *P<0.05, **P<0.01, ***P<0.001.

Supplementary Figure 3

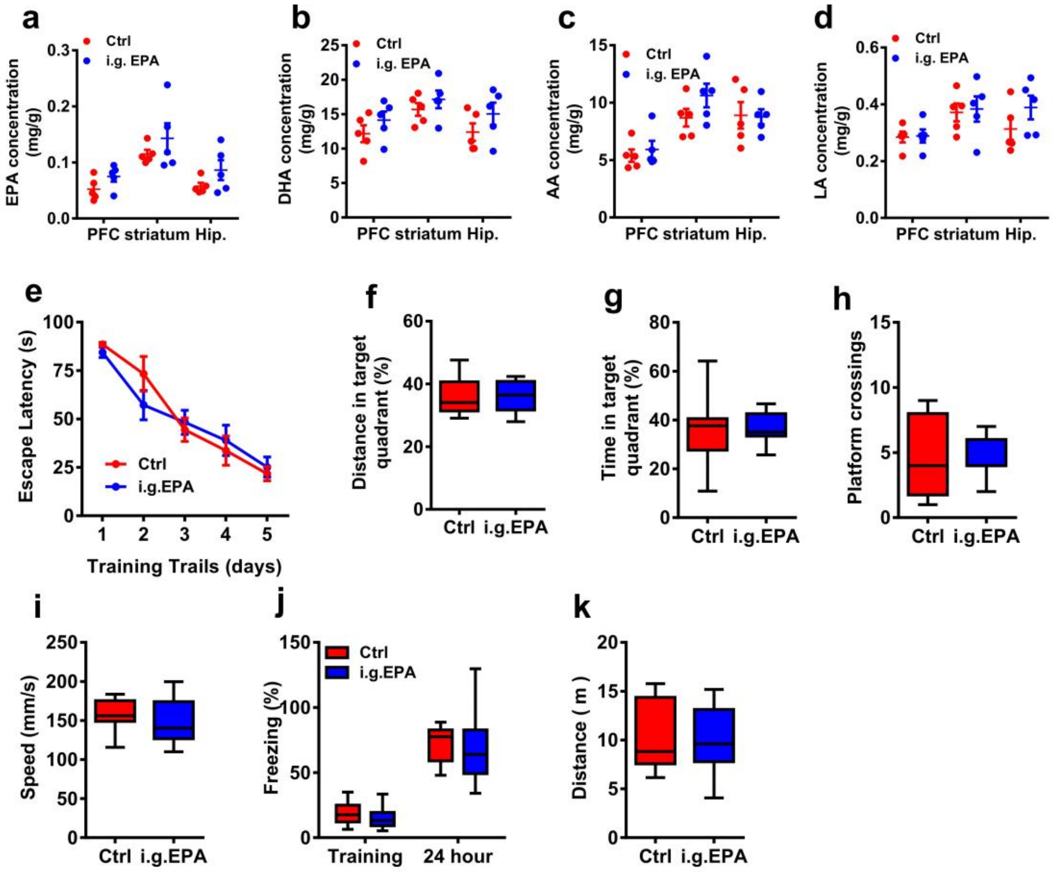

Supplementary Figure 3

Effects of chronic administration of EPA on fatty acids levels and learning and memory behavior.

(**a-d**) The levels of EPA, DHA, AA, and LA in the brain were not affected 24 hours after the last day of i.g. administration of EPA that lasted one month (50 mg/kg, n =5 mice/group).

(**e-k**) EPA did not impair the learning and memory 24 hours after the last day of i.g. administration of EPA (50 mg/kg) that lasted one month in MWM (n=10,12 mice; **e**: repeated measures two-way ANOVA, $F_{(1, 90)}$=30.146, P=0.512. **f-i**: two-tailed Student's t-test; **f**: P=0.754; **g**: P=0.466; **h**: P=0.541; **i**: P=0.754) and contextual fear conditioning test (**j**: n=9,10 mice; two-tailed Student's t-test, P=0.215) without affecting the locomotor activity in the open field test (**k**: n=9,10 mice; two-tailed Student's t-test, P=0.642).

Data show mean ± s.e.m..

Supplementary Figure 4

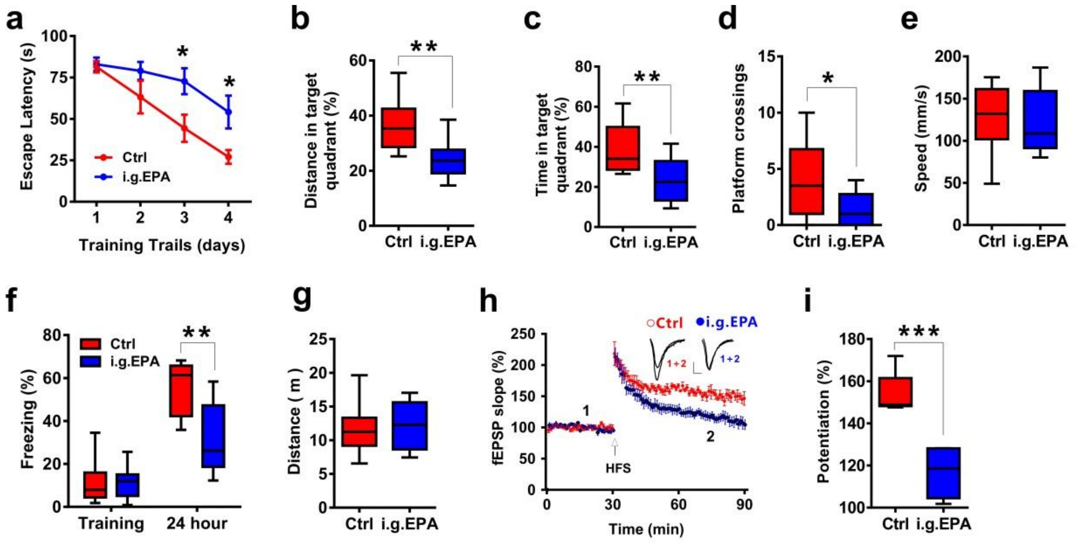

Supplementary Figure 4

EPA administration impaired learning and memory and LTP in prepubescent mice (P21).

(**a**) Mean escape latencies during the MWM training after i.g. administration of EPA (50 mg/kg, n=12 mice/group; repeated measures two-way ANOVA, $F_{(1, 88)}$=58.309, P=0.96$\times 10^{-3}$). (**b**, **c**) Percentage of distance (**b**) or time (**c**) spent in the target quadrant during the probe trials (n=12 mice/group; two-tailed Student's t-test; **b**: P=0.001; **c**: P=0.004).

(**d**) Number of platform crossings during the probe trials (n=12 mice/group; two-tailed Student's t-test, P=0.021).

(**e**) Swimming speed in the MWM test (n=12 mice/group; two-tailed Student's t-test, P=0.788).

(**f**) Freezing time in the contextual fear conditioning test after i.g. administration of EPA (50 mg/kg, n=12 mice/group; two-tailed Student's t-test, P=0.002).

(**g**) Locomotor activity in the open field test (n=12 mice/group; two-tailed Student's t-test, P=0.324).

(**h**, **i**) HFS-LTP after i.g. administration of EPA (50 mg/kg, n=5,7 slices; two-tailed Student's t-test, P=0.76$\times 10^{-4}$). Scale bars: 0.5 mV, 5 ms.

Data show mean ± s.e.m.. *P<0.05, **P< 0.01, ***P< 0.001.

Supplementary Figure 5

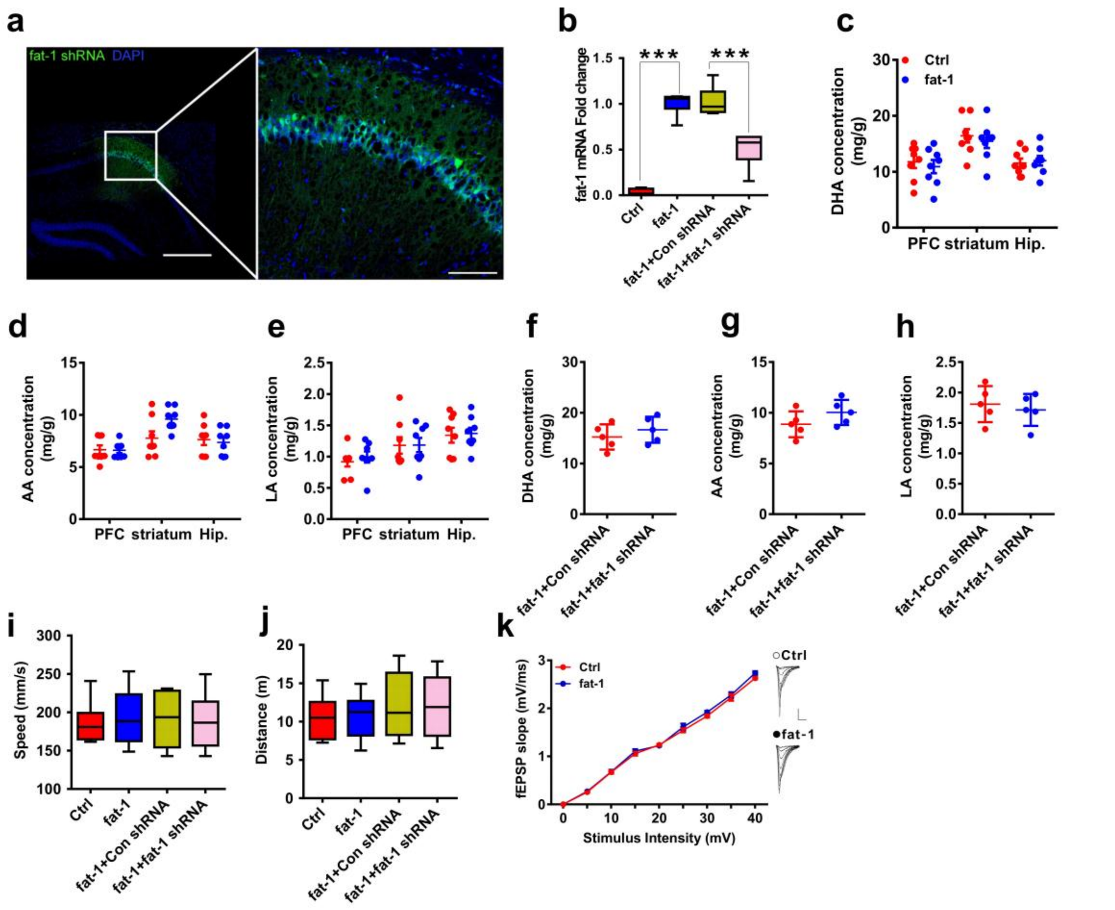

Supplementary Figure 5

Effects of knocking down the fat-1 gene on fatty acid levels, behaviors and synaptic transmission.

(**a**) Representative fluorescence images showing the locations and cells infected with the pAAV-CAG-fat-1-EGFP (shRNA) vector in the CA1 region of fat-1 mice. Scale bar: left: 500 µm, right: 100 µm. Experimental images were obtained from seven mice; three images per mouse were taken, with similar results obtained.

(**b**) Level of fat-1 mRNA in fat-1 mice with or without injecting the shRNA (n=6 mice/group; two-tailed Student’s t-test; Ctrl vs fat-1: P<0.0001, fa-1+con shRNA vs fat-1+fat-1 shRNA: $P=0.66\times10^{-3}$).

(**c**-**e**) Levels of DHA, AA, and LA in the brain region of fat-1 mice (n=8 mice/group).

(**f**-**h**) Levels of DHA, AA, and LA in the brain region of fat-1 mice with injecting the shRNA (n=5 mice/group).

(**i, j**) Swimming speed in the MWM (i, n=10,9,9,9 mice; one-way ANOVA, $F_{(3, 33)}$=20.06, P=0.98) and locomotor activity in open field test (j, n=10,12,9,9 mice; one-way ANOVA, $F_{(3, 36)}$=20.459, P=0.688) in fat-1 mice with or without shRNA and their control littermates.

(**k**) I-O curves in fat-1 mice and their control littermates (n=6 slices/group; repeated measures two-way ANOVA, $F_{(1,90)}$=13.739, P=0.745). Scale bars: 0.5 mV, 5 ms.

Data show mean ± s.e.m. ***P< 0.001.

Supplementary Table 1

receptor	Radioligand (nM)	non-ligand (μM)	EPA (50μM) I%	DHA (100μM) I%
**5-HT$_{1A}$**	**[3H]-8-OH-DPAT(2)**	**way100635(10)**	**0**	**0**
**5-HT$_{1B}$**	**[3H]-LSD(6)**	**5-HT(10)**	**12**	**10**
**5-HT$_{1D}$**	**[3H]-LSD(10)**	**5-HT(10)**	**14**	**11**
**5-HT$_{2A}$**	**[3H]-spiperone(1)**	**Clozapine（10）**	**9**	**4**
**5-HT$_{2C}$**	**[3H]-LSD(4)**	**5-HT(10)**	**4**	**70**
**5-HT$_{5A}$**	**[3H]-LSD(1)**	**5-HT(10)**	**19**	**0**
**5-HT$_{6}$**	**[3H]-LSD(1)**	**5-HT(10)**	**64**	**12**

Supplementary Table 1

Radio-ligand Receptor Binding Assay

EPA selectively bound to the 5-HT$_6$R as its high I% compared to that bound to other 5-HT receptors, while DHA selectively bound to the 5-HT$_{2C}$R (EPA, eicosapentaenoic acid; DHA, docosahexaenoic acid; 5-HT, 5-Hydroxytryptamine; 8-OH-DPAT, (±)-8-Hydroxy-2-(dipropylamino) tetralinhydrobromide, selective 5-HT$_1$A receptor agonist; WAY100635, selective 5-HT$_1$A receptor antagonist; LSD, Lysergic acid diethylamide, a non-selective serotonin receptor agonist; spiperone, 5-HT$_2$A receptor agonist; Clozapine, 5-HT$_2$A receptor antagonist).

Supplementary Figure 6

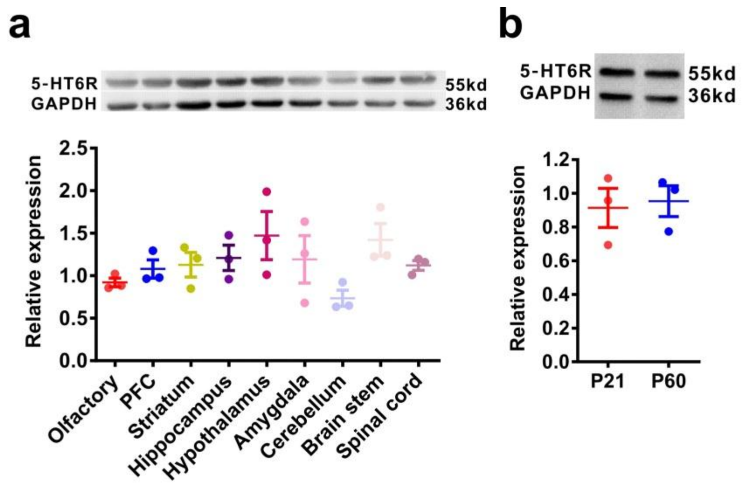

Supplementary Figure 6

The expression level of 5-HT$_6$R in different brain regions and ages.

(**a**) Western blots of 5-HT$_6$R from different brain regions of adult mice (n=3 experiments/group; one-way ANOVA, $F_{(8, 21)}$=37.543, P=0.057).

(**b**) Western blots of 5-HT$_6$R in the hippocampus from adult and prepubescent mice (n=3 experiments/group; two-tailed Student’s t-test; P=0.801).

Full-length blots for **a** and **b** are presented in Supplementary Figure 9. Data show mean ± s.e.m.

Supplementary Figure 7

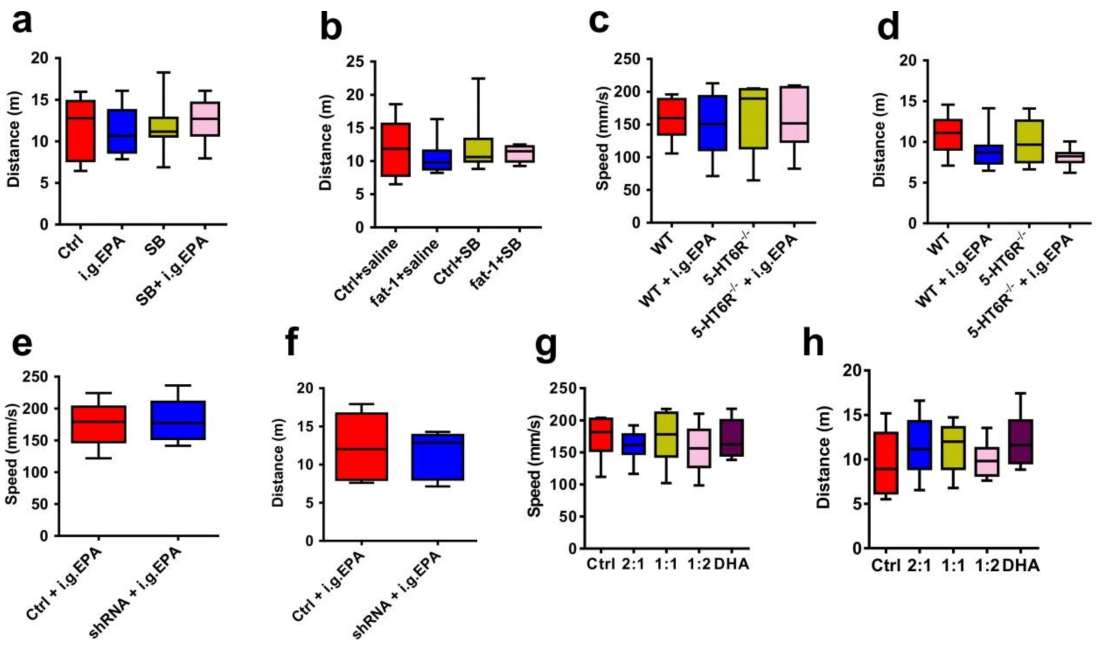

Supplementary Figure 7

The locomotor activity and swimming speed after different treatment.

(**a, b**) Locomotor activity in the open field test after CA1 microinjection of SB (300 nM) in i.g. EPA (50mg/kg)-treated mice (**a**, n=9,11,10,10 mice; one-way ANOVA, $F_{(3, 36)}$=11.540, P=0.489) or fat-1 mice (**b**, n=9,12,8,9 mice; one-way ANOVA, $F_{(3, 34)}$=22.128, P=0.109).

(**c, d**) Swimming speed in the MWM (**c**, n=10,12,10,11 mice; one-way ANOVA, $F_{(3, 39)}$=23.71, P=0.157)) and locomotor activity in the open field test (**d**, n=10,12,10,11 mice; one-way ANOVA, $F_{(3, 39)}$=13.159, P=0.24) for 5-HT$_6$R KO mice with or without i.g. EPA.

(**e, f**) Effects of i.g. EPA on the swimming speed in the MWM (**e**, n=9,8 mice; two-tailed Student's t-test, P=0.794) and the locomotor activity in the open field test (**f**, n=10,11 mice; two-tailed Student's t-test, P=0.474) in GAD-Cre mice with or without injecting the shRNA.

(**g, h**) Swimming speed in the MWM test (g, n=10,10,10,11,11 mice; one-way ANOVA, $F_{(4, 47)}$=13.558, P=0.43) and locomotor activity in the open field test (h, n=10,10,10,11,11 mice; one-way ANOVA; $F_{(4, 47)}$=10.635, P=0.831) in mice that treated with EPA/DHA at different ratio.

Data show mean ± s.e.m.

Supplementary Figure 8

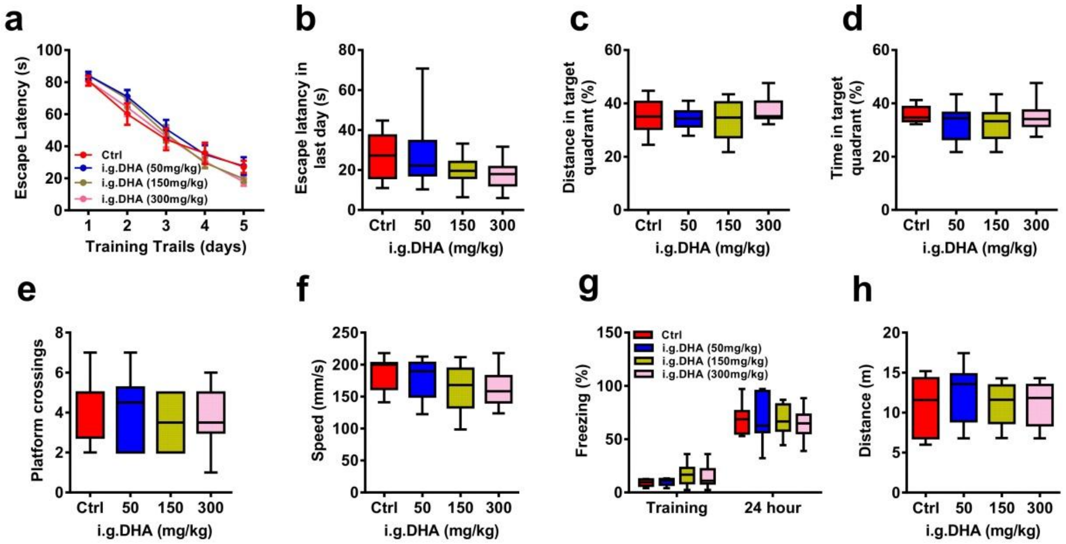

Supplementary Figure 8

DHA administration did not affect learning and memory in adult mice (P60).

(**a, b**) Mean escape latencies across 5 consecutive days (**a**) or on the last day (**b**) during the MWM training after i.g. administration of DHA (n=9,10,10,10 mice; **a**: repeated measures two-way ANOVA, $F_{(3, 175)}$=18.309, P=0.764; **b**: one-way ANOVA, $F_{(3, 35)}$=23.806, P=0.16).

(**c**, **d**) Percentage of distance (**c**) or time (**d**) spent in the target quadrant during the probe trials (n=9,10,10,10 mice; one-way ANOVA; **c**: $F_{(3, 35)}$=14.256, P=0.616; **d**: $F_{(3, 35)}$=12.461, P=0.718).

(**e**) Number of platform crossings during the probe trials (n=9,10,10,10 mice; one-way ANOVA, $F_{(3, 35)}$=15.044, P=0.926).

(**f**) Swimming speed in the MWM test (n=9,10,10,10 mice; one-way ANOVA, $F_{(3, 35)}$=14.932, P=0.584).

(**g**) Freezing time in the contextual fear conditioning test after i.g. administration of EPA (n=9,10,10,10 mice; one-way ANOVA, $F_{(3, 35)}$=21.456, P=0.678).

(**h**) Locomotor activity in the open field test (n=9,10,10,10 mice; one-way ANOVA, $F_{(3, 35)}$=17.678, P=0.608).

Data show mean ± s.e.m..

Supplementary Figure 9

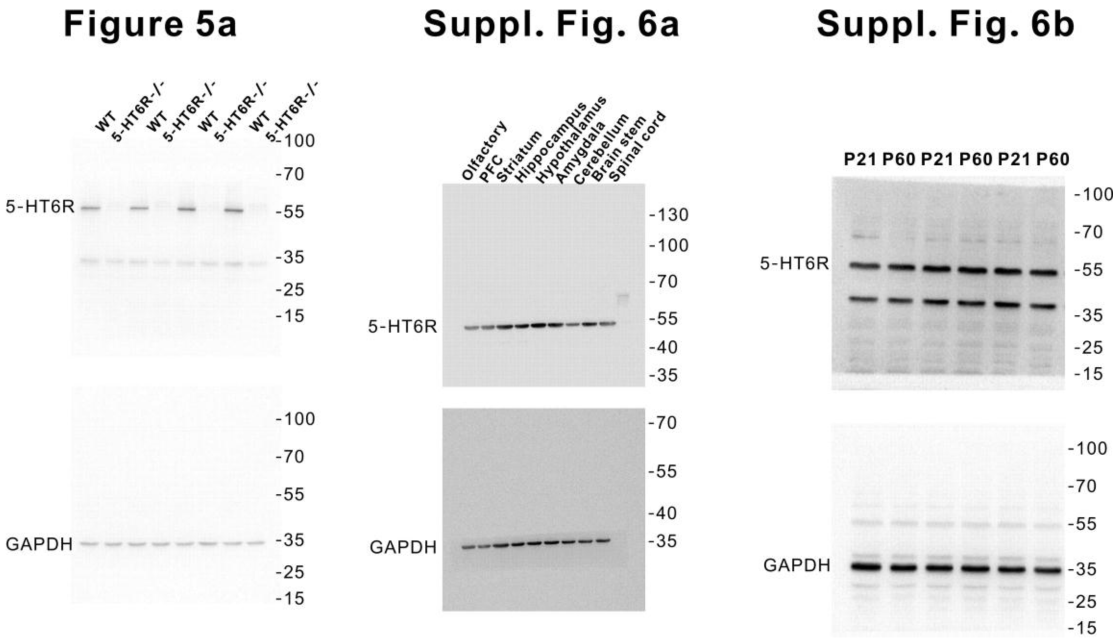

Supplementary Figure 9

Full-length pictures of the blots presented in the main figures and supplementary figures.
