# Supplementary material for: Acute EPA-induced learning and memory impairment in mice is prevented by DHA
Source: Nat Commun. 2020 Oct 29;11:5465. doi: 10.1038/s41467-020-19255-1 (PMC7596714; doi:10.1038/s41467-020-19255-1)
Supplement: Supplementary file 3 — Reporting Summary [file 41467_2020_19255_MOESM3_ESM.pdf]

nature research

Corresponding author(s): T.M. Gao

Last updated by author(s): Sep 29, 2020

Reporting Summary

Nature Research wishes to improve the reproducibility of the work that we publish. This form provides structure for consistency and transparency in reporting. For further information on Nature Research policies, see our Editorial Policies and the Editorial Policy Checklist.

Statistics

For all statistical analyses, confirm that the following items are present in the figure legend, table legend, main text, or Methods section.

n/a	Confirmed	
☐	☒	The exact sample size (n) for each experimental group/condition, given as a discrete number and unit of measurement
☐	☒	A statement on whether measurements were taken from distinct samples or whether the same sample was measured repeatedly
☐	☒	The statistical test(s) used AND whether they are one- or two-sided<br>*Only common tests should be described solely by name; describe more complex techniques in the Methods section.*
☐	☒	A description of all covariates tested
☐	☒	A description of any assumptions or corrections, such as tests of normality and adjustment for multiple comparisons
☐	☒	A full description of the statistical parameters including central tendency (e.g. means) or other basic estimates (e.g. regression coefficient) AND variation (e.g. standard deviation) or associated estimates of uncertainty (e.g. confidence intervals)
☐	☒	For null hypothesis testing, the test statistic (e.g. F, t, r) with confidence intervals, effect sizes, degrees of freedom and P value noted<br>*Give P values as exact values whenever suitable.*
☒	☐	For Bayesian analysis, information on the choice of priors and Markov chain Monte Carlo settings
☐	☒	For hierarchical and complex designs, identification of the appropriate level for tests and full reporting of outcomes
☐	☒	Estimates of effect sizes (e.g. Cohen's d, Pearson's r), indicating how they were calculated

Our web collection on statistics for biologists contains articles on many of the points above.

Software and code

Policy information about availability of computer code

Data collection	Ethovision XT 7 software (Noldus) was used for animal tracking. Observer 5.0 (Noldus) was used for behavioural scoring. Adobe Illustrator CC (2014) for assembling figures; Adobe After Effects CC for video rendering; GraphPad Prism version 5.0 (GraphPad Software) was used to draw figures; Multiclamp 700B (Axon Instrument, Molecular Devices) was used to electrophysiological recordings.
Data analysis	GC Chemstation Revision B.04.03 Driver Service Pack 1 (DSP1) was used to analyzed the fatty acids composition; pClamp 10v. 10.6.2.2 (Molecular Devices) was used to analyzed electrophysiological results; Statistical analyses were performed in SPSS 20.0 software.

For manuscripts utilizing custom algorithms or software that are central to the research but not yet described in published literature, software must be made available to editors and reviewers. We strongly encourage code deposition in a community repository (e.g. GitHub). See the Nature Research guidelines for submitting code & software for further information.

Data

Policy information about availability of data

All manuscripts must include a data availability statement. This statement should provide the following information, where applicable:

- Accession codes, unique identifiers, or web links for publicly available datasets
- A list of figures that have associated raw data
- A description of any restrictions on data availability

The source data underlying Figs. 1–8 and Supplementary Figs. 1–8 are provided as source data files. The rest of relevant data are available from the corresponding author on reasonable request.

Field-specific reporting

Please select the one below that is the best fit for your research. If you are not sure, read the appropriate sections before making your selection.

[x] Life sciences [ ] Behavioural & social sciences [ ] Ecological, evolutionary & environmental sciences

For a reference copy of the document with all sections, see nature.com/documents/nr-reporting-summary-flat.pdf

Life sciences study design

All studies must disclose on these points even when the disclosure is negative.

Sample size	No statistical methods were used to predetermine sample size, but our sample sizes are similar to those reported in previous publications (e.g. Bicks et at, 2020, Sun et al 2016)
Data exclusions	Mice were excluded from the study if their viral expression could not be validated or when there was equipment failure during behavior, or if they demonstrated significant alterations in motor responses due to i.p. procedures.
Replication	For all studies, independent animals were used as replicates. Replication studies confirmed our results in all experiments.
Randomization	Animals and eukaryotic cell were randomly assigned numbers and tested blind for the experimental condition.
Blinding	All behavioral experiments were scored by an individual blind to the genotype and experimental design. And Data collection and analysis were not performed in a blinded fashion due to the nature of the experiments.

Reporting for specific materials, systems and methods

We require information from authors about some types of materials, experimental systems and methods used in many studies. Here, indicate whether each material, system or method listed is relevant to your study. If you are not sure if a list item applies to your research, read the appropriate section before selecting a response.

Materials & experimental systems

n/a	Involved in the study	
[ ]	[x]	Antibodies
[ ]	[x]	Eukaryotic cell lines
[x]	[ ]	Palaeontology and archaeology
[ ]	[x]	Animals and other organisms
[x]	[ ]	Human research participants
[x]	[ ]	Clinical data
[x]	[ ]	Dual use research of concern

Methods

n/a	Involved in the study	
[x]	[ ]	ChIP-seq
[x]	[ ]	Flow cytometry
[x]	[ ]	MRI-based neuroimaging

Antibodies

Antibodies used	Primary antibodies: rabbit anti-5-HT6R (Abcam, ab103016) 1:1,000, mouse anti-GAD67 (Millipore, MAB5406) 1:500. Secondary antibodies (all from TInvitrogen): Alexa Fluor 488 (A11034) 1:500, Alexa Fluor 594 (A11005) 1:500.
Validation	Data sheet and website of Synaptic systems describes that anti-5-HT6R antibody and anti-GAD67 antibody were validated to react specifically with mouse 5-HT6R and GAD67, respectively and has been used and cited by over 40 papers. The secondary antibodies were also validated and has been used and cited by over 40 papers.

Eukaryotic cell lines

Policy information about cell lines

Cell line source(s)	ATCC: 293T
Authentication	ATCC authentication involving detection of species specific variants of the cytochrome C oxidase I gene (COI analysis; to confirm human origin) and short tandem repeat profiling (to rule out intra-species contamination).
Mycoplasma contamination	RNAseq analysis identified no mycoplasma contamination.
Commonly misidentified lines (See ICLAC register)	No commonly misidentified cell lines were used in the study.

Animals and other organisms

Policy information about studies involving animals; ARRIVE guidelines recommended for reporting animal research

Laboratory animals	Male mice of the following strains were used, at 3 weeks or 8-12 weeks of age: C57BL/6J mice (provided by Guangzhou Southern Medical University Animal Center); 5-HT6R−/− mice (purchased from the Mutant Mouse Resource & Research Center); fat-1 transgenic mice (donated by Professor Xiaochun Bai, Southern Medical University); GAD-Cre mice (purchased from the Mutant Mouse Resource & Research Center). Mice were housed in standard laboratory cages (4–5 per cage), with a 12-h light/dark cycle (lights on at 8:00 A.M.), in a temperature-controlled room (21–25 °C). Mice were provided free access to food and water.
Wild animals	The study did not involve the wild animals.
Field-collected samples	The study did not involve samples collected from the field.
Ethics oversight	All experiments were conducted in accordance with the Regulations for the Administration of Affairs Concerning Experimental Animals (China), and were approved by the Southern Medical University Animal Ethics Committee.

Note that full information on the approval of the study protocol must also be provided in the manuscript.
